# An immobilized Schiff base–Mn complex as a hybrid magnetic nanocatalyst for green synthesis of biologically active [4,3-*d*]pyrido[1,2-*a*]pyrimidin-6-ones†

**DOI:** 10.1039/d4na00131a

**Published:** 2024-04-04

**Authors:** Mohammad Ali Bodaghifard, Seied Ali Pourmousavi, Najmieh Ahadi, Payam Zeynali

**Affiliations:** a Department of Chemistry, Faculty of Science, Arak University Arak 384817758 Iran m-bodaghifard@araku.ac.ir mbodaghi2007@yahoo.com; b Institute of Nanosciences &Nanotechnology, Arak University Arak Iran; c School of Chemistry, Damghan University Damghan 36716-45667 Iran

## Abstract

The immobilization of metal ions on inorganic supports has garnered significant attention due to its wide range of applications. These immobilized metal ions serve as catalysts for chemical reactions and as probes for studying biological processes. In this study, we successfully prepared Fe_3_O_4_@SiO_2_@Mn-complex by immobilizing manganese onto the surface of magnetic Fe_3_O_4_@SiO_2_ nanoparticles through a layer-by-layer assembly technique. The structure of these hybrid nanoparticles was characterized by various analytical techniques, including Fourier transform infrared spectroscopy (FT-IR), powder X-ray diffraction (PXRD), thermogravimetric analysis (TGA), vibrating sample magnetometry (VSM), scanning electron microscopy (SEM), and inductively coupled plasma-optical emission spectrometry (ICP-OES). Fe_3_O_4_@SiO_2_@Mn-complex was successfully utilized in the synthesis of biologically active 7-aryl[4,3-*d*]pyrido[1,2-*a*]pyrimidin-6(7*H*)-one derivatives in an aqueous medium, providing environmentally friendly conditions. The desired products were manufactured in high yields (81–95%) without the formation of side products. The heterogeneity of the solid nanocatalyst was assessed using a hot filtration test that confirmed minimal manganese leaching during the reaction. This procedure offers numerous advantages, including short reaction times, the use of a green solvent, the ability to reuse the catalyst without a significant decrease in catalytic activity, and easy separation of the catalyst using an external magnet. Furthermore, this approach aligns with environmental compatibility and sustainability standards.

## Abstract 2

Fe_3_O_4_@SiO_2_@Mn-complex was successfully prepared and utilized in the synthesis of biologically active pyridopyrimidinones in an aqueous medium, providing environmentally friendly conditions.

## Graphical abstract

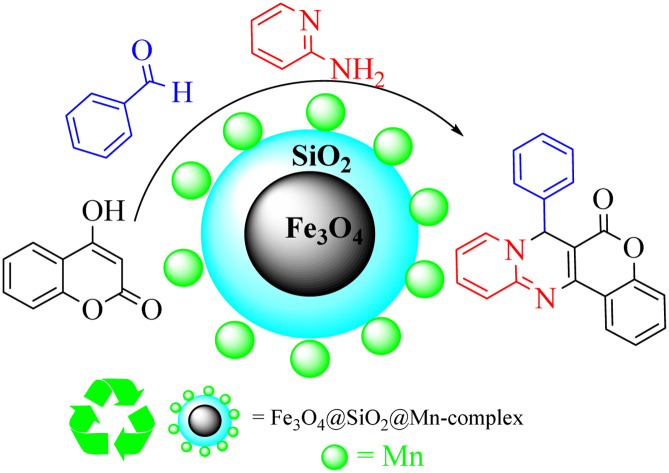

## Introduction

Nanotechnology is poised to revolutionize various technological applications, with nanomaterials playing a pivotal role in numerous fields such as pharmacology, medicine, environmental protection, electronics, and particularly catalysis.^1^ Nanocatalysts demonstrate superior catalytic activity compared to conventional catalysts due to their high surface area. Additionally, they possess remarkable chemical stability.^2^ In recent years, magnetic nanoparticles (MNPs) have gained significant popularity as a support for homogeneous catalysts. MNP-supported catalysts offer several noteworthy features. First, the use of MNPs as a support enhances the stability and recyclability of the catalyst. The magnetic nature of MNPs enables the catalyst to be easily separated and recovered from reaction mixtures by applying an external magnetic field. This simplifies the purification process and reduces waste generation, making MNP-supported catalysts more environmentally friendly. Additionally, MNP-supported catalysts exhibit high surface area due to their small particle size, which promotes increased catalytic activity. The large surface area facilitates better interaction between the reactants and the catalyst, leading to improved reaction rates and higher conversion efficiencies. Moreover, MNP-supported catalysts can be easily functionalized with various organic ligands or metal complexes, allowing for tailoring their catalytic properties to specific reactions.^3–5^

Spinel ferrites (MFe_2_O_4_, M: Fe, Zn, Co, Ni, Cd, *etc.*), with their unique properties and wide range of applications, hold significant importance in the field of magnetic materials. These materials exhibit high saturation magnetization and coercivity values, making them highly suitable for a wide range of magnetic applications. The utilization of ferrite spheres with a hollow structure extends to various domains, including catalysis, adsorbents, and gas sensors. They find extensive use in electronic devices,^6^ information storage,^7^ magnetic resonance imaging (MRI),^8^ drug-delivery technology,^9^ adsorption,^10^ and sensing applications.^11^ Moreover, they can serve as catalysts in various chemical processes.^12,13^ The hollow interior provides a large surface area, allowing for enhanced contact between the catalyst and reactants. Furthermore, the hollow structure allows for easy diffusion of reactants and products within the sphere, facilitating faster reaction rates. Consequently, these spheres have become a focal point of interest among researchers.^7^ Magnetite, also known as iron(ii, iii) oxide (Fe_3_O_4_), is a unique type of ferrite that can serve as an excellent magnetic support material for catalysts. It possesses high saturation magnetization, low cost, high chemical stability, and mechanical strength.^8,14,15^ The high saturation magnetization allows for easy separation of the catalyst from the reaction mixture using an external magnetic field, simplifying the recovery and recycling process. The low cost of magnetite makes it economically viable for large-scale industrial applications. Metal complexes have been utilized as homogeneous catalysts in a wide array of reactions. However, many of these complexes are costly and precious, and they come with drawbacks such as difficult separation and a decline in catalytic activity over time. To address these challenges, researchers have focused on immobilizing metal complexes onto inorganic supports.^16–18^ There have been numerous studies on the grafting and immobilization of metal complexes onto magnetic nanosized inorganic supports, demonstrating significant benefits in terms of enhanced catalytic activity and stability.^19–27^ Supported metal complexes offer the advantage of easy separation from reaction mixtures using an external magnetic field, making them highly efficient and practical for catalytic applications. Furthermore, immobilizing metal complexes on inorganic supports can improve their catalytic performance by creating a stable environment for active sites, leading to improved selectivity and reusability. Furthermore, the development of supported metal complexes on magnetic nanosized inorganic supports represents a promising strategy for overcoming the limitations of traditional homogeneous catalysts and advancing sustainable catalysis processes.

Multicomponent reactions (MCRs) have revolutionized the field of organic synthesis by enabling the rapid and efficient construction of complex molecules in a single step, saving time and resources.^28,29^ By utilizing different combinations of starting materials, catalysts, and reaction conditions, researchers can access a wide range of structurally diverse compounds with potential biological activities or unique properties. Moreover, MCRs align well with the principles of green chemistry. Green chemistry aims to minimize environmental impact by designing chemical processes that are sustainable, efficient, and safe. MCRs contribute to this goal by reducing waste generation through atom economy, ensuring that most atoms from the starting materials end up in the final product. This flexibility makes MCRs highly valuable in drug discovery, materials science, and other fields where access to structurally diverse compounds is crucial.^30,31^

**Scheme 1:**
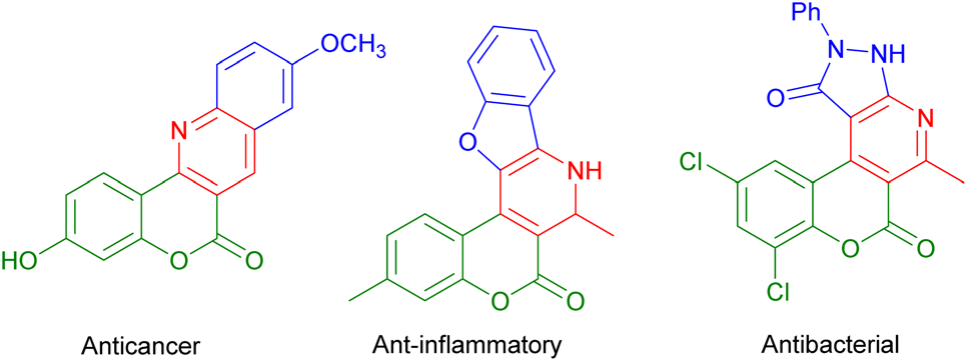
Biologically active fused coumarin derivatives.

Polyfunctionalized heterocyclic moieties are commonly found in the structures of bioactive natural and synthetic lead molecules, as well as in drug candidates that are either already on the market or currently undergoing clinical trials.^32,33^ These versatile compounds are also prevalent in agrochemicals, cosmetics, dyes, and various other application-oriented materials.^34^ The abundance of polyfunctionalized heterocyclic moieties in these compounds underscores their importance and potential for further exploration. Natural coumarins and their synthetic analogues are widely recognized for their diverse range of significant pharmacological and biological properties within O-heterocycles. Coumarin and its derivatives are highly versatile molecules that exhibit a broad spectrum of biological activities, including anti-inflammatory, antifungal, antimicrobial, anticancer, antiviral, antitumor, antioxidant, and antidiabetic properties.^34–37^ (Scheme 1) These compounds have immense potential for therapeutic applications in various fields of medicine and research.^37,38^ One particular class of coumarin derivatives, chromeno[4,3-*d*]pyrido[1,2-*a*]pyrimidin-6-ones, stands out due to its distinctive fused ring system. The fusion of chromene, pyridine, and pyrimidine rings results in a three-dimensional structure that confers specific chemical and biological activities. These compounds exhibit remarkable potential for various applications in the fields of medicine and biology.^39–42^

**Scheme 2:**
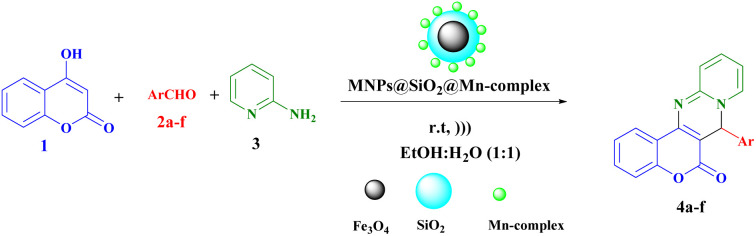
Synthesis of chromeno[4,3-*d*]pyrido[1,2-*a*]pyrimidin-6-ones in the presence of Fe_3_O_4_@SiO_2_@Mn-complex.

Water, as a green solvent, offers several advantages in organic synthesis. First, it is abundant, inexpensive, and readily available, making it a sustainable choice for large-scale reactions.^43^ Additionally, water is non-toxic and non-flammable, ensuring the safety of both researchers and the environment.^44,45^ The ability of water to form hydrogen bonds with substrates plays a crucial role in accelerating organic reactions. Hydrogen bonding facilitates the dissolution of reactants and enhances their reactivity by stabilizing transition states.^46,47^ The development of environmentally friendly and sustainable synthetic methodologies has gained significant attention in recent years.^48,49^ In line with this, a new organic–inorganic hybrid nanostructure (Fe_3_O_4_@SiO_2_@Mn-complex) was prepared by immobilizing manganese onto the surface of magnetic Fe_3_O_4_@SiO_2_ nanoparticles through a layer-by-layer assembly technique. This hybrid nanostructure efficiently catalysed the green synthesis of pharmaceutically interesting chromeno pyridopyrimidines under aqueous conditions (Scheme 2).

## Experimental

All chemicals were purchased from Merck Chemical Company. Melting points were determined using ElectroThermal 9100 apparatus. The progress of the reaction was followed by thin-layer chromatography (TLC) on UV active aluminum backed plates of silica gel (TLC Silica gel 60 F254). ^1^H and ^13^C NMR spectra were recorded on a Bruker Advance spectrometer at 300 and 75 MHz, respectively in DMSO-*d*_6_ with TMS as an internal standard. IR spectra were reported using a Bruker Alpha using KBr pellets in the range of 400–4000 cm^−1^. X-ray diffraction (XRD) was carried out on an X Pert Pro Cu-Kα radiation (*λ* = 0.15406 nm) in the range of Bragg angle 10–80°. The field emission-scanning electron microscopy (FE-SEM) and energy-dispersive X-ray spectroscopy (EDS) analyses were performed on a Sigma system, Zeiss Company, Germany. Thermal stability of Fe_3_O_4_@SiO_2_@Mn-complex (Fe_3_O_4_@SiO_2_@Mn-complex) MNPs was studied by thermogravimetric analysis (TGA) on a TGA2 system, Mettler Toledo Company under an N_2_ atmosphere at a heating rate of 10 °C min^−1^. The magnetic properties of MNPs were studied using a vibration sample magnetometer (VSM) with the model of an LBKFB instrument (Meghnatis Daghigh Kavir).

### Preparation of the magnetic Fe_3_O_4_ nanoparticles

Fe_3_O_4_ MNPs were prepared by the co-preparation method.^14^ A mixture of FeCl_2_·4H_2_O (5 mmol, 0.99 g), and FeCl_3_·6H_2_O (10 mmol, 2.7 g) and deionized water (50 mL) in a round bottom flask (100 mL) was heated for 1 h at 95 °C under a N_2_ atmosphere. Then pH of the reaction was adjusted on pH = 10 using ammonium hydroxide (25%) and heated for another 1 h. The reaction mixture was cooled to room temperature and the obtained black precipitate was separated using a magnet. The precipitate was washed several times with warm deionized water and ethanol (20 mL) and dried in a vacuum oven at 70 °C.

### Synthesis of silica-coated MNPs (Fe_3_O_4_@SiO_2_ MNPs)

Fe_3_O_4_ MNPs were coated with a silica layer using the Stöber method.^50^ A mixture of Fe_3_O_4_ MNPs (1 g), EtOH (99%, 4 mL) and deionized water (6 mL) was poured in a round bottom flask (100 mL) and sonicated for 20 minutes. Ammonium hydroxide (25%, 1.5 mL) was added to the solution and sonicated for 20 minutes again. Tetraethyl orthosilicate (TEOS) (1.4 mL) was added to solution and stirred for 12 h under a N_2_ atmosphere at room temperature. The black sediment was separated from the reaction using a magnet and washed with deionized water and ethanol several times and dried in a vacuum oven at 70 °C.

### Synthesis of Fe_3_O_4_@SiO_2_–PrNH_2_ nanoparticles

Fe_3_O_4_@SiO_2_ MNPs (1 g) were dispersed under sonication in dry toluene (10 mL) for 20 minutes. Then, 3-aminopropyltrimethoxysilane (APTMS) (0.6 mL) was added and refluxed for 24 h under a N_2_ atmosphere. Finally, the precipitate was separated using a magnet, washed with toluene (10 mL), and dried in a vacuum oven at 50 °C.^51^

### Preparation of Fe_3_O_4_@SiO_2_@2OH-1NAP nanoparticles

A mixture of Fe_3_O_4_@SiO_2_–PrNH_2_ MNPs (1 g) and EtOH (10 mL) was sonicated for 20 minutes. Then, 2-hydroxy-1-naphthaldehyde (0.3 g, 1.75 mmol) and triethylamine as base (0.8 mL) were added to the solution and refluxed for 24 h under a N_2_ atmosphere. The reaction mixture was cooled to room temperature. The sediment was isolated using an external magnet and washed with ethanol three times (15 mL) and dried in a vacuum oven at 50 °C.

### Preparation of the hybrid magnetic nanostructure (Fe_3_O_4_@SiO_2_@Mn-complex)

Fe_3_O_4_@SiO_2_@2OH-1NAP MNPs (0.5 g) were dispersed in EtOH (10 mL) for 20 minutes. Then, MnCl_2_·4H_2_O solution (2 M, 5 mL) was added to the mixture slowly for 1 hour and stirred for 24 h under a N_2_ atmosphere at room temperature. The precipitate was separated using a magnet, washed with EtOH and H_2_O, and dried in a vacuum oven at 50 °C.

### General procedure for the synthesis of chromeno[4,3-*d*]pyrido[1,2-*a*]pyrimidin-6-one derivatives

Fe_3_O_4_@SiO_2_@Mn-complex MNPs (15 mg) were added to a mixture of aromatic aldehydes (1 mmol), 2-aminopyridine (1 mmol) and 4-hydroxycoumarin (1 mmol) in EtOH : H_2_O (1 : 1) (6 mL). The mixture was stirred under ultrasonic waves at room temperature and the progress of the reaction was monitored by thin layer chromatography (TLC). After completion of the reaction, the catalyst was separated from the reaction using a magnet. 5 mL of water was added to the solution to form the product. The obtained precipitate was filtered and dried at room temperature.

### Selected spectral data

#### 7-(4-Chlorophenyl)-6*H*,7*H*-chromeno[4,3-*d*]pyrido[1,2-*a*]pyrimidin-6-one (4a, Fig. S1 and S2†)

White powder, mp 230–232 °C;^40^^1^H NMR (300 MHz, DMSO-*d*_6_): *σ* (ppm) = 8.23–6.66 (m, 12H, HAr), 5.33 (s, 1H, CH). ^13^C NMR (75 MHz, DMSO-*d*_6_): *σ* (ppm) = 168.2, 165.5, 152.9, 149.3, 142.3, 141.8, 141.3, 139.7, 131.4, 129.7, 128.9, 128.0, 124.5, 123.3, 120.1, 115.9, 112.7, 103.7, 36.2.

#### 7-(3,4-Dimethoxyphenyl)-6*H*,7*H*-chromeno[4,3-*d*]pyrido[1,2-*a*]pyrimidin-6-one (4h, Fig. S5 and S6†)

Light purple, mp 210–212 °C;^39^^1^H NMR (300 MHz, DMSO-*d*_6_): *σ* (ppm) = 3.44 (s, 3H, –OCH_3_), 3.61 (s, 3H, –OCH_3_), 6.11 (s, 1H, CH), 6.55–6.59 (m, 2H, HAr), 6.68 (d, 1H, *J* = 8.22 Hz, aromatic), 7.14–7.20 (m, 5H, HAr), 7.44 (t, 1H, *J* = 8.6 Hz, aromatic), 7.74 (d, 1H, *J* = 7.74 Hz, HAr). ^13^C-NMR (75 MHz, DMSO-*d*_6_): *σ* (ppm) = 168.9, 166.9, 156.5, 152.9, 150.4, 143.6, 141.2, 134.8, 134.6, 133.8, 131.6, 130.9, 129.4, 128.7, 122.9, 121.7, 121.1, 113.1, 110.7, 105.7, 58.1, 54.7, 36.9.

#### 7-(4-Hydroxy-3-methoxyphenyl)-6*H*,7*H*-chromeno[4,3-*d*]pyrido[1,2-*a*]pyrimidin-6-one (4j, Fig. S7 and S8†)

Light yellow, mp 175–177 °C;^39^^1^H NMR (300 MHz, DMSO-*d*_6_): *σ* (ppm) = 3.77 (s, 3H, –OCH_3_), 6.17 (s, 1H, CH), 6.50 (s, 1H, HAr), 6.59 (s, 1H, HAr), 6.97–7.49 (m, 7H, HAr), 7.81 (s, 2H, HAr), 8.46 (brs, 1H, OH). ^13^C-NMR (75 MHz, DMSO-*d*_6_): *σ* (ppm) = 168.1, 166.3, 153.7, 152.9, 147.7, 146.4, 144.9, 133.5, 131.3, 129.2, 126.3, 124.5, 123.3, 120.5, 119.8, 115.9, 115.4, 113.8, 112.3, 104.3, 64.3, 36.1.

#### 7-(2-Hydroxyphenyl)-6*H*,7*H*-chromeno[4,3-*d*]pyrido[1,2-*a*]pyrimidin-6-one (4l, Fig. S9 and S10†)

Yellow, mp 165–166 °C; ^1^H NMR (300 MHz, DMSO-*d*_6_): *σ* (ppm) = 6.17 (s, 1H, CH), 6.59 (d, 1H, *J* = 7.59 Hz, HAr), 6.76 (d, 1H, *J* = 8.39 Hz, HAr), 7.14 (d, 1H, *J* = 7.41 Hz, HAr), 7.22 (d, 4H, *J* = 7.68 Hz, HAr), 7.43 (t, 3H, *J* = 7.23 Hz, HAr), 7.67 (t, 1H, *J* = 7.32 Hz, HAr), 7.81 (d, 1H, *J* = 7.68 Hz, HAr), 8.35 (brs, 1H, OH). ^13^C-NMR (75 MHz, DMSO-*d*_6_): *σ* (ppm) = 167.6, 164.7, 155.5, 152.8, 149.8, 147.1, 141.4, 130.9, 129.6, 129.4, 126.4, 124.4, 123.1, 120.7, 118.2, 115.7, 115.1, 112.5, 111.5, 104.2, 33.3.

#### 7-(3-Nitrophenyl)-6*H*,7*H*-chromeno[4,3-*d*]pyrido[1,2-*a*]pyrimidin-6-one (4m, Fig. S11 and S12†)

Light yellow, mp 275–277 °C; ^1^H NMR (300 MHz, DMSO-*d*_6_): *σ* (ppm) = 6.35 (s, 1H, CH), 6.55–6.82 (m, 2H, HAr), 7.27 (s, 2H, HAr), 7.54 (s, 2H, HAr), 7.82–7.98 (m, 3H, HAr), 8.34–8.83 (m, 2H, HAr). ^13^C-NMR (75 MHz, DMSO-*d*_6_): *σ* (ppm) = 168.5, 164.9, 153.0, 148.7, 148.2, 145.5, 137.7, 135.6, 134.3, 131.9, 129.9, 129.1, 124.7, 124.5, 123.7, 121.5, 120.9, 120.1, 116.1, 103.1, 36.7.

## Results and discussion

### Preparation and characterization of the magnetic hybrid nanostructure (Fe_3_O_4_@SiO_2_@Mn-complex)

**Scheme 3:**
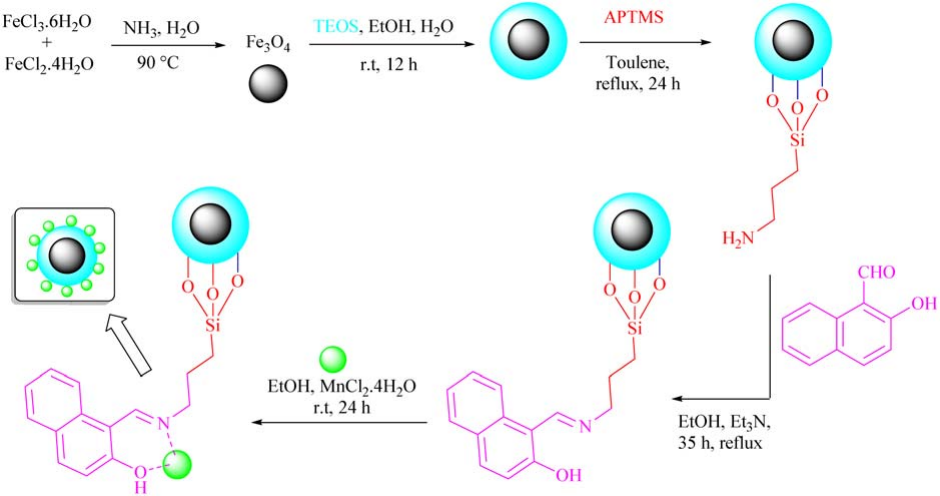
The pathway for preparation of hybrid MNPs (Fe_3_O_4_@SiO_2_@Mn-complex).

Fe_3_O_4_@SiO_2_@Mn-complex was constructed following the procedure outlined in Scheme 3. Initially, Fe_3_O_4_ magnetic nanoparticles (MNPs) were prepared using the co-precipitation method. Subsequently, these particles were coated with a layer of silica using the Stöber method, resulting in the formation of Fe_3_O_4_@SiO_2_ MNPs. The Fe_3_O_4_@SiO_2_–PrNH_2_ nanoparticles were fabricated by functionalization of Fe_3_O_4_@SiO_2_ nanoparticles with APTMS. In continuation, a Schiff base ligand was constructed on the surface of MNPs *via* the condensation of NH_2_ groups with 2-hydroxynaphthaldehyde. Finally, Mn was stabilized and immobilized on modified MNPs through the coordination of nitrogen and oxygen atoms. The resulting precipitate was washed with ethanol and dried in a vacuum oven to obtain the final hybrid nanomaterial (Fe_3_O_4_@SiO_2_@Mn-complex). Various characterization techniques including FT-IR spectroscopy, X-ray diffraction (XRD), field emission scanning electron microscopy (FE-SEM), energy dispersive X-ray spectroscopy (EDS), thermogravimetric analysis (TGA), vibrating sample magnetometry (VSM), and inductively coupled plasma-optical emission spectrometry (ICP-OES) were used to identify and characterize the prepared nanostructure.

**Fig. 1:**
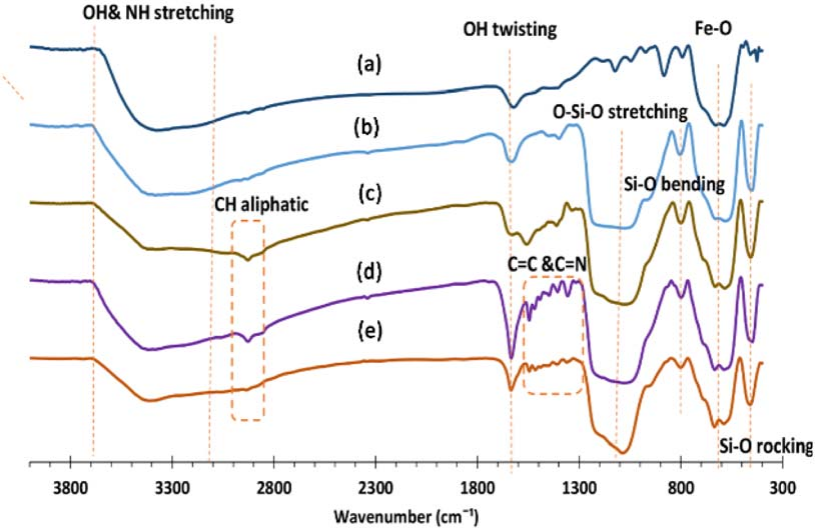
FT-IR spectra of Fe_3_O_4_ (a), Fe_3_O_4_@SiO_2_ (b), Fe_3_O_4_@SiO_2_@PrNH_2_ (c), Fe_3_O_4_@SiO_2_–PrNH@2OH-1NAP (d), and Fe_3_O_4_@SiO_2_@Mn-complex (e) MNPs.

FT-IR spectra of Fe_3_O_4_ (a), Fe_3_O_4_@SiO_2_ (b), Fe_3_O_4_@SiO_2_@PrNH_2_ (c), Fe_3_O_4_@SiO_2_–PrNH@2OH-1NAP (d), and Fe_3_O_4_@SiO_2_@Mn-complex (e) are presented in Fig. 1. A broad absorption band is observed in the range of 3000 and 3500 cm^−1^, which can be related to the stretching vibrations of the OH and NH groups (Fig. 1a–d). In addition, the absorption band at 1623 cm^−1^ is related to the OH twisting vibration band (Fig. 1a), which is shifted to 1634 cm^−1^ due to the modification of NPs (Fig. 1e). The Fe–O vibration bands appeared at 628 and 588 cm^−1^ (Fig. 1a–d). The appearance of absorption bands at 1078, 969, 803 and 448 cm^−1^ is attributed to the asymmetric stretching, symmetric stretching, in plane bending and rocking modes of the Si–O–Si group, respectively, that confirm the formation of a SiO_2_ shell (Fig. 1b). Weak absorption bands are observed at around 2970 cm^−1^, which confirm the presence of aliphatic hydrogens after modification using APTMS (Fig. 1c). In addition, a new band at 1558 cm^−1^ proves the existence of bending NH (Fig. 1c). The appearance of new absorption bands in the range of 1356–1545 cm^−1^ can confirm the C=N and C=C bonds in the nanostructure (Fig. 1d and e). Finally, in Fig. 1e, no significant change was observed, and therefore the presence of manganese in the nanoparticle structure was considered by other techniques. In general, the results prove that the surface of nanoparticles has been successfully modified.

**Fig. 2:**
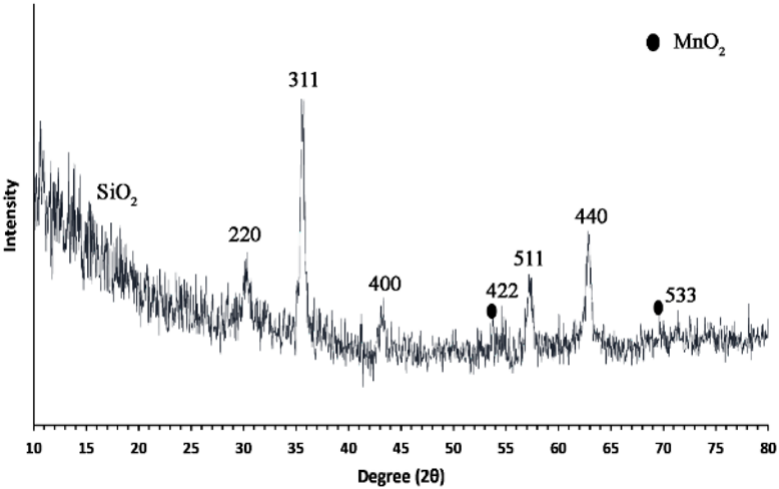
The XRD pattern of the Fe_3_O_4_@SiO_2_@Mn-complex nanostructure.

The XRD pattern of Fe_3_O_4_@SiO_2_@Mn-complex NPs is shown in Fig. 2. The sample was well ground using a rotary motion with a mortar and pestle to obtain a finer powder and better data. The diffraction peaks in Bragg's angles 2*θ* = 30.25°, 35.68°, 43.32°, 55.05°, 57.31°, 62.89°, and 74.99° correspond to the 220, 311, 400, 422, 511, 440 and 533 planes of Fe_3_O_4_, respectively. These data indicate that the obtained Fe_3_O_4_ nanoparticles have a spinel structure and are according to the standard JCPDS (file no. 0629-19).^52^ Also, the formation of peaks at 2*θ* = 70.10° and 53.55° can confirm the presence of MnO_2_ in the nanostructure.^53^

**Fig. 3:**
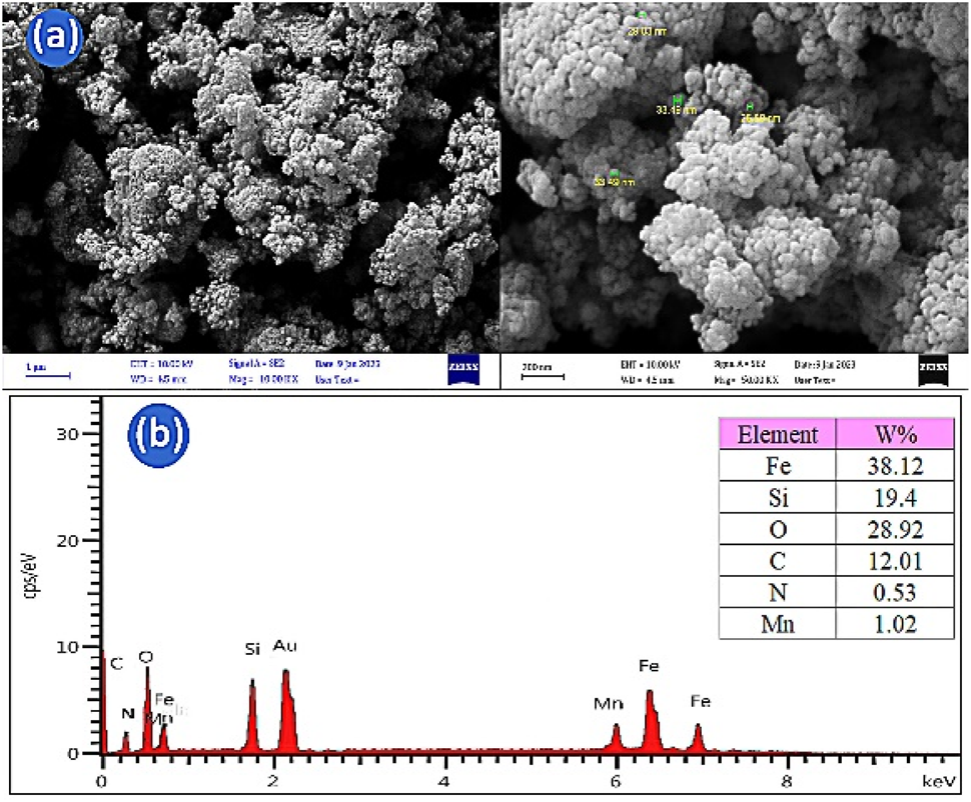
FE-SEM images (a) and EDX analysis (b) of the Fe_3_O_4_@SiO_2_@Mn-complex nanostructure.

The peak of SiO_2_ is observed in the range of 2*θ* = 17–20°.^54^ The XRD patterns prove that the structure of Fe_3_O_4_ nanoparticles has been preserved during the functionalization steps. The crystal size was calculated using Scherer's equation (*D* = 0.9*λ*/*β* cos *θ*). In this equation, *λ* is the wavelength of copper (0.154 nm), *θ* is the Bragg's angle of the peak with the highest intensity (35.68°), and *β* is half the height of the peak with the highest intensity. The crystallite size of MNPs calculated from the width of the peak at 2*θ* = 35.68°(311) is 15 nm, which is smaller than the range determined using FE-SEM analysis (Fig. 3).

**Fig. 4:**
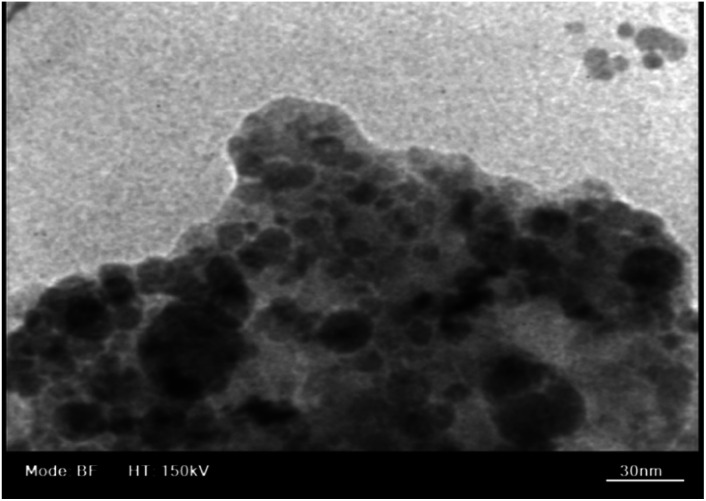
TEM image of the Fe_3_O_4_@SiO_2_@Mn-complex nanostructure.

Scanning electron microscopy (FE-SEM) was applied to check the morphology and size of nanoparticles (Fig. 3a). As can be seen, the prepared nanoparticles have average diameters of 25–35 nm and spherical shapes. The size of nanoparticles obtained from FE-SEM images is larger than that obtained from the XRD patterns, which can be attributed to some accumulation of nanoparticles. The EDX analysis shows the presence of iron (Fe), silicon (Si), oxygen (O), carbon (C), nitrogen (N) and manganese (Mn) elements in the Fe_3_O_4_@SiO_2_@Mn-complex nanostructure (Fig. 3b). Furthermore, the higher intensity of the Si peak compared to the Fe peaks shows that the Fe_3_O_4_ nanoparticles have been trapped by the SiO_2_ layer. The black spots and grey parts in the TEM image correspond to the Fe_3_O_4_ core and silica layer (Fig. 4). Based on the TEM images, the sizes of the catalyst particles were found to be less than 40 nm. The typical thickness of the silica shell was evaluated to be in the range of 5–20 nm. The SEM and TEM images reveal that the size of the catalyst particles falls within the nanometer range.

**Fig. 5:**
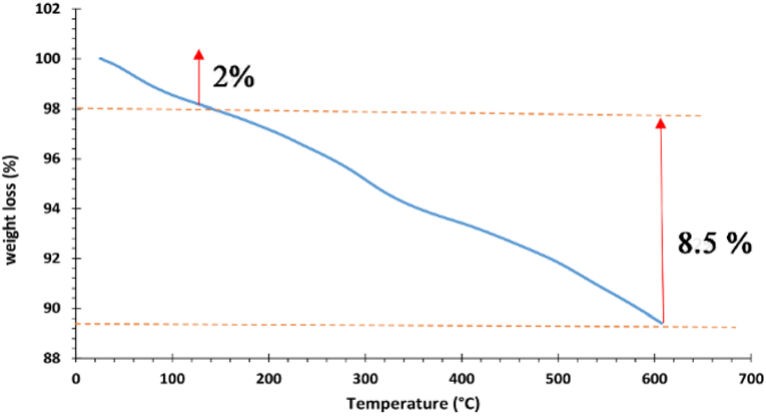
TGA analysis of Fe_3_O_4_@SiO_2_@Mn-complex MNPs.

The thermogravimetric analysis (TGA) was used to study the thermal stability of Fe_3_O_4_@SiO_2_@Mn-complex at 50–600 °C (Fig. 5). The magnetic catalyst shows 14% weight loss in two main steps over the temperature range. The first stage shows 2% weight loss at *T* < 150 °C, which can be related to the removal of physically adsorbed water and organic solvents. The second stage shows 9% weight loss in the range of 150 < *T* < 600 °C, which is attributed to the decomposition of the organic moieties grafted on the nanostructure. The exact amount of manganese in Fe_3_O_4_@SiO_2_@Mn-complex was measured using the ICP-OES technique. Based on ICP-OES analysis, the amount of manganese in the hybrid nanocatalyst was 1.93 × 10^−3^ mol g^−1^.

**Fig. 6:**
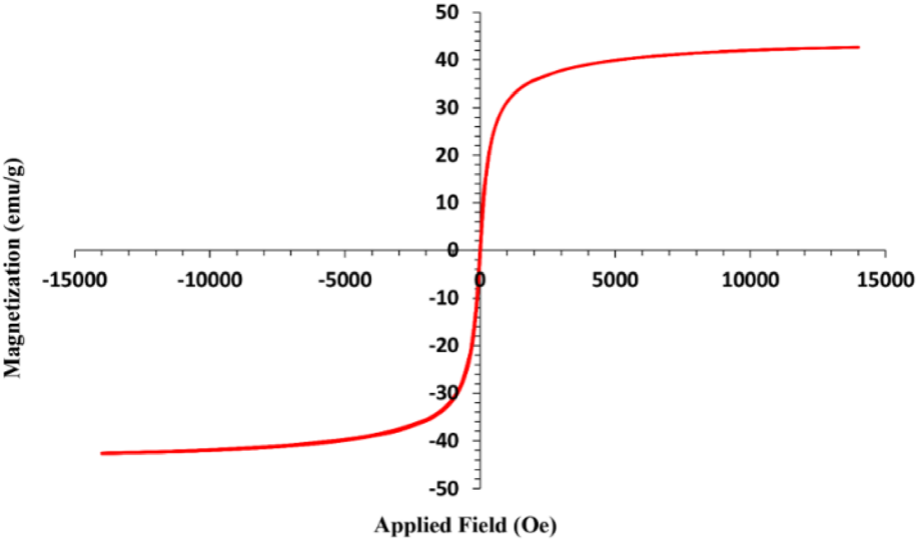
VSM analysis of Fe_3_O_4_@SiO_2_@Mn-complex MNPs.

Vibrating sample magnetometry was carried out to investigate the magnetic properties of the prepared NPs in a ±15 000 Oe range at room temperature (Fig. 6). The hysteresis loop is S like and shows superparamagnetic behavior for magnetic nanoparticles and no hysteresis phenomenon was observed. The histogram curve provides the main information about saturation magnetization (*M*_s_). As can be seen, the nanoparticles have shown a saturation magnetization (*M*_s_) of about 42 emu g^−1^ that confirms that Fe_3_O_4_@SiO_2_@Mn-complex MNPs have a suitable *M*_s_ after modification during four steps yet. So, this heterogeneous catalyst can be separated easily from the solution by using an external magnet.

### Catalytic activity of Fe_3_O_4_@SiO_2_@Mn-complex in the synthesis of chromeno[4,3-*d*]pyrido[1,2-*a*]pyrimidin-6-ones

After synthesis and characterization of the Fe_3_O_4_@SiO_2_@Mn-complex nanostructure, its catalytic activity was investigated in the synthesis of chromeno[4,3-*d*]pyrido[1,2-*a*]pyrimidin-6-one derivatives. The condensation reaction of 4-hydroxycoumarin isatin, 4-chlorobenzaldehyde, and 2-aminopyridine was selected as the model reaction. Three important parameters: solvents, catalyst amount, and temperature were optimized and the results are summarized in Table 1. First, the model reaction was carried out in several solvents such as EtOH, H_2_O, EtOH : H_2_O, CH_3_CN, and DMF and under solvent-free conditions. It was found that EtOH : H_2_O (1 : 1) is the best solvent for this reaction (Table 1, entry 4). As can be seen in Table 1, increasing the amount of catalyst from 5 to 15 mg improved the reaction time and efficiency, but, increasing the amount of catalyst to 20 mg did not have a significant effect on the reaction time and efficiency (Table 1, entries 7–9). Finally, the reaction was examined at temperatures ranging from room temperature to 70 °C (Table 1, entries 3, 10, 11). The use of 15 mg of Fe_3_O_4_@SiO_2_@Mn-complex as a catalyst in EtOH : H_2_O (1 : 1) as a solvent under ultrasound sonication serves as the best condition with respect to the green nature, clean workup procedure, and high yield for this synthesis. To consider the role of Fe_3_O_4_@SiO_2_@Mn-complex as a catalyst, the model reaction was performed under the same conditions with Fe_3_O_4_, Fe_3_O_4_@SiO_2_@PrNH@2OH-1NAP and without any catalyst (Table 1, entries 11–13). The findings confirm that the catalytic activity of Fe_3_O_4_@SiO_2_@Mn-complex was improved in comparison to Fe_3_O_4_, and Fe_3_O_4_@SiO_2_@PrNH@2OH-1NAP. Immobilizing Mn metal on the nanostructure improves the reaction rate and product yield.

**Table 1:** The optimization of reaction conditions for synthesis of chromeno[4,3-*d*]pyrido[1,2-*a*]pyrimidin-6-one derivatives a

Entry	Catalyst	Solvent	Temp. (°C)	Time (min)	Yield^b^ (%)
1	MNPs@Mn-complex (5 mg)	—	r.t	320	60
2	MNPs@Mn-complex (5 mg)	H_2_O	r.t	120	75
3	MNPs@Mn-complex (5 mg)	EtOH	r.t	100	78
4	MNPs@Mn-complex (5 mg)	H_2_O/EtOH (1 : 1)	r.t	90	80
5	MNPs@Mn-complex (5 mg)	CH_3_CN	r.t	480	—
6	MNPs@Mn-complex (5 mg)	DMF	r.t	480	—
7	MNPs@Mn-complex (10 mg)	H_2_O/EtOH (1 : 1)	r.t	70	83
8	MNPs@Mn-complex (15 mg)	H_2_O/EtOH (1 : 1)	r.t	40	95
9	MNPs@Mn-complex (20 mg)	H_2_O/EtOH (1 : 1)	r.t	40	95
10	MNPs@Mn-complex (15 mg)	H_2_O/EtOH (1 : 1)	50	40	93
11	MNPs@Mn-complex (15 mg)	H_2_O/EtOH (1 : 1)	70	40	94
12	Fe_3_O_4_ (15 mg)	H_2_O/EtOH (1 : 1)	r.t	60	65
13	MNPs@PrNH@2OH-1NAP (15 mg)	H_2_O/EtOH (1 : 1)	r.t	60	40
14	—	H_2_O/EtOH (1 : 1)	r.t	120	10

a 4-Hydroxycoumarin (1 mmol), 4-chlorobenzaldehyde (1 mmol) and 2-aminopyridine (1 mmol), and Fe_3_O_4_@SiO_2_@Mn-complex.

b Isolated yield.

The heterogeneity of the solid nanocatalyst was assessed by passing the model reaction mixture through a preheated filter pad during the hot filtration test. The catalytically active nanoparticles were separated from the reaction by filtration after 20 minutes. The filtered reaction solution was then allowed to react for 60 minutes under optimal conditions. Reaction analysis and metal measurement in solution indicated minimal manganese leaching during the reaction and a significant decrease in the reaction rate following the hot filtration process.

After optimizing the reaction conditions, various aromatic aldehydes with electron-withdrawing or electron-donating substituents were applied under the optimized reaction conditions to determine the efficacy of the catalyst (Scheme 2 and Table 2). For all substrates, the reaction proceeded efficiently to produce the desired chromeno[4,3-*d*]pyrido[1,2-*a*]pyrimidin-6-one derivatives in high to excellent yields with short reaction times without the formation of side products. The catalytic activity of Fe_3_O_4_@SiO_2_@Mn-complex MNPs for the model reaction was also compared with that reported in the literature (Table 3). These data confirm that the Fe_3_O_4_@SiO_2_@Mn-complex nanostructure is a suitable catalyst for synthesizing the desired corresponding product.

**Table 2:** Synthesis of chromeno[4,3-*d*]pyrido[1,2-*a*]pyrimidin-6-one derivatives under optimal conditions^a^

Entry	ArCHO	Product	Time (min)	Yield^b^ (%)	Mp (°C)	
					Obtained	Reported^39–41^
1	4-Cl–C_6_H_4_CHO	4a	40	95	233–235	236–238
2	C_6_H_5_CHO	4b	40	93	203–204	206–208
3	4-CH_3_–C_6_H_4_CHO	4c	40	91	228–230	231–233
4	4-NO_2_–C_6_H_4_CHO	4d	30	95	221–222	225–226
5	4-Br–C_6_H_4_CHO	4e	30	94	237–238	240–242
6	4-N(CH_3_)_2_–C_6_H_4_CHO	4f	40	87	190–192	182–183
7	4-OCH_3_–C_6_H_4_CHO	4g	40	90	211–212	206–207
8	3,4-(OCH_3_)_2_–C_6_H_3_CHO	4h	45	89	210–212	215–216
9	2,4-Cl_2_–C_6_H_3_CHO	4i	30	93	144–146	136–138
10	3-OCH_3_–4-OH–C_6_H_3_CHO	4j	40	87	181–182	189–190
11	2-Cl–C_6_H_4_CHO	4k	40	85	235–237	238–240
12	2-OH–C_6_H_4_CHO	4l	50	81	165–166	New
13	3-NO_2_–C_6_H_4_CHO	4m	50	91	275–277	New
14	3-Cl–C_6_H_4_CHO	4n	40	93	177–178	179–181

a 4-Hydroxycoumarin (1 mmol), 4-chlorobenzaldehyde (1 mmol) and 2-aminopyridine (1 mmol), Fe_3_O_4_@SiO_2_@Mn-complex (15 mg) under ultrasound waves, EtOH : H_2_O (1 : 1) at room temperature.

b Isolated yield.

**Table 3:** Comparison of catalytic activity of Fe_3_O_4_@SiO_2_@Mn-complex with those in reported studies for 4a

Entry	Catalyst	Conditions	Time (min)	Yield (%)	Ref.
1	NH_2_SO_3_H	r.t, EtOH : H_2_O (1 : 1)	35 min	91	39
2	[CMMIM][BF_4_^−^]	r.t, EtOH : H_2_O (1 : 1)	3.5 h	92	40
3	NiFe_2_O_4_@SiO_2_ grafted di(3-propylsulfonic acid)	EtOH (50%), reflux, 80 °C	5 h	89	41
4	Fe_3_O_4_@SiO_2_@Mn-complex	r.t, EtOH : H_2_O (1 : 1)	40 min	95	This work

**Scheme 4:**
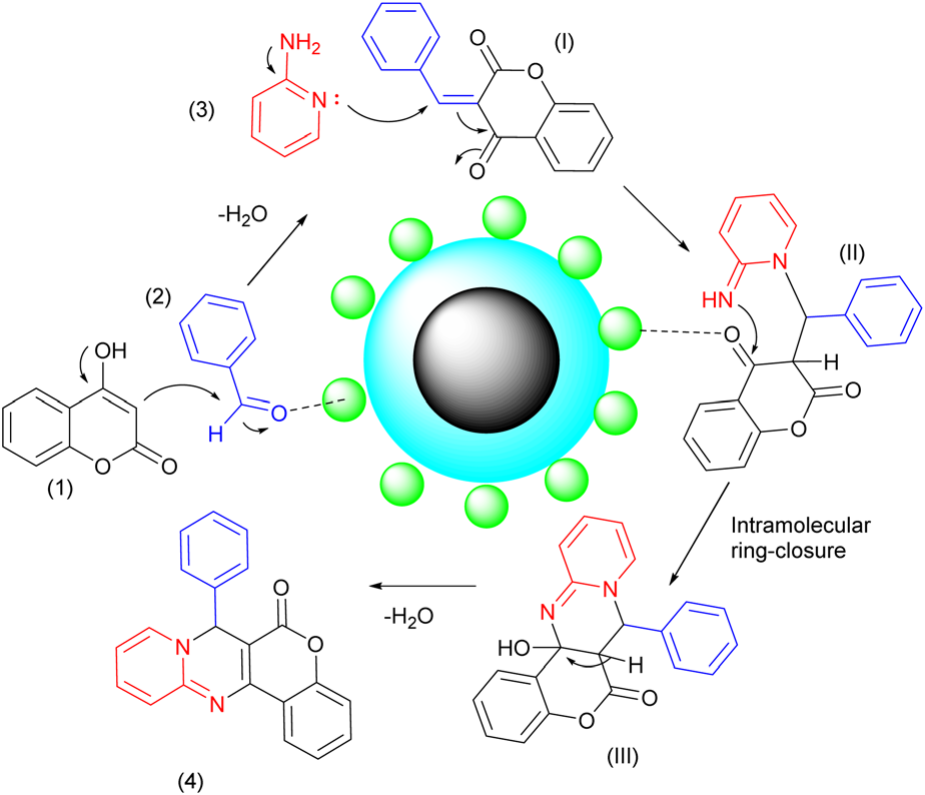
A plausible mechanism for the synthesis of chromeno[4,3-*d*]pyrido[1,2-*a*]pyrimidin-6-ones in the presence of the Fe_3_O_4_@SiO_2_@Mn-complex nanostructure.

A plausible mechanism for the reaction is proposed in Scheme 4. The process is initiated by the Lewis acid sites of the nanostructure activating the carbonyl group of the aldehyde. This activation leads to the formation of intermediate (I) through a condensation reaction between 4-hydroxycoumarin (1) and the aldehyde (2), resulting in the elimination of a water molecule. Next, 2-aminopyridine (3) performs a nucleophilic attack on intermediate (I), which then undergoes a keto–enol tautomerization to form intermediate (II). To complete the reaction, intermediate (II) undergoes a simple intramolecular ring closure. The desired product resulted from the removal of a water molecule.^39^ The nanostructure (Fe_3_O_4_@SiO_2_@Mn-complex) possesses high porosity and surface area, which enhances the adsorption and activation of reactant molecules and provides a suitable support for this chemical reaction. Moreover, the presence of Lewis acid sites within the nanostructure can also activate certain bonds within the reactant molecules, making them more susceptible to breaking or rearranging during the reaction process. These activation steps effectively lower the energy barrier for the reactions to occur, accelerating the reaction rates.

**Fig. 7:**
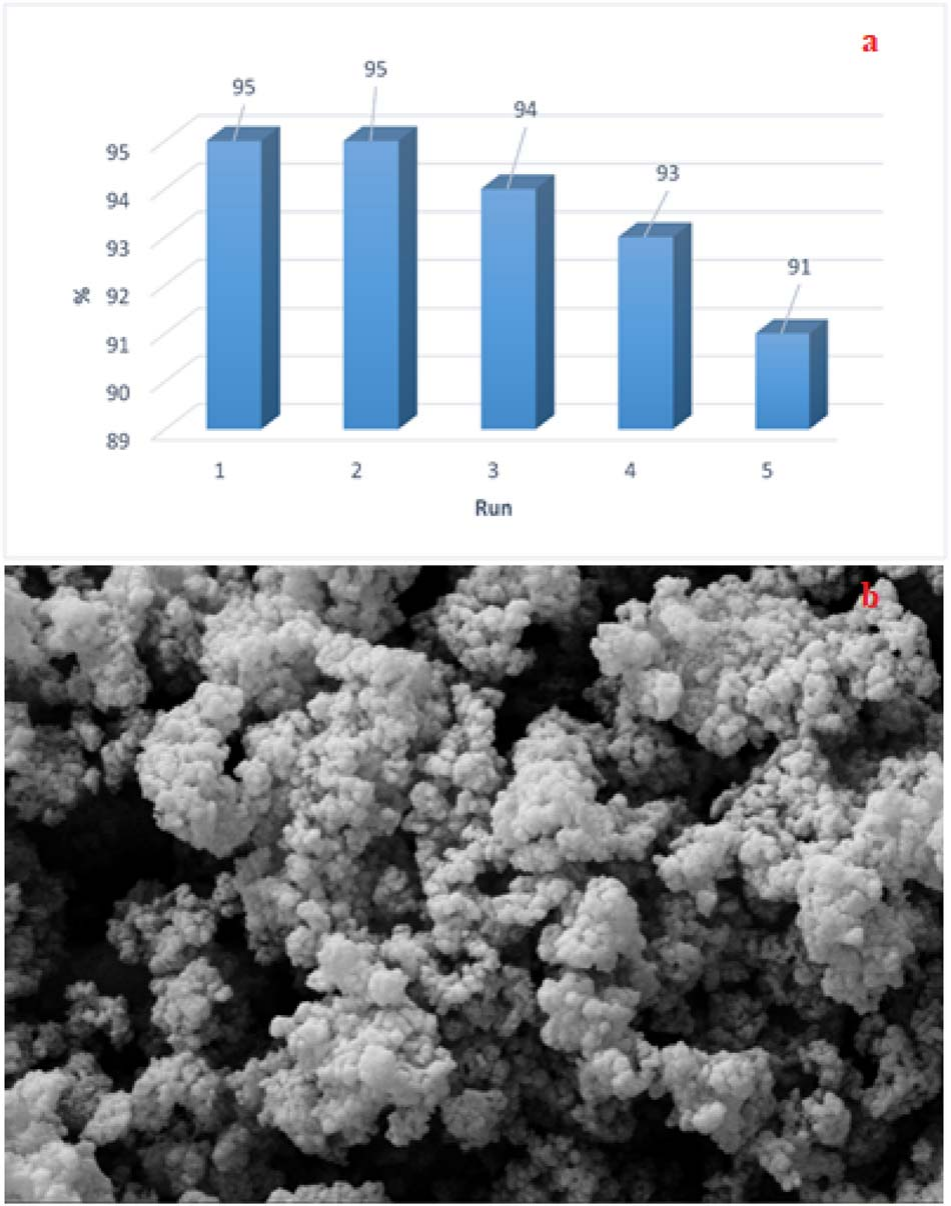
(a) Recyclability of Fe_3_O_4_@SiO_2_@Mn-complex in the model reaction during four runs. (b) FE-SEM image of the reused nanocatalyst.

The recyclability and reusability properties of the Fe_3_O_4_@SiO_2_@Mn-complex nanostructure were investigated under optimal conditions in the model reaction. After completion of each reaction, the catalyst was easily separated using an external magnet, washed with ethanol, dried in a vacuum oven, and reused in subsequent reactions. As shown in Fig. 7, the recycled catalyst could be reused at least four times without any additional treatment or appreciable reduction in catalytic activity and without any change in its morphology. The product yield decreased by only 4% after four runs, indicating the high performance of the catalyst. Nearly, quantitative recovery of the catalyst (up to 95%) could be obtained from each run. The consistent structure and activity of the recovered catalyst (Fe_3_O_4_@SiO_2_@Mn-complex) confirm that the reused nanocatalyst exhibits excellent performance in the synthesis of chromeno[4,3-*d*]pyrido[1,2-*a*]pyrimidin-6-one derivatives. We attribute this success to the complexation of Mn with organic ligands and immobilization on an inorganic support that effectively limits the leaching and particle growth of Mn. This unique hybrid nanocatalyst can be reused many times with only a slight decrease in performance.

ICP-OES analysis was used to measure the potential Mn leaching into the reaction mixture after four runs. After four cycles, the manganese content was determined to be 1.87 × 10^−3^ mol g^−1^. The manganese content in Fe_3_O_4_@SiO_2_@Mn-complex after four runs was comparable to that in the fresh catalyst (1.93 × 10^−3^ mol g^−1^), confirming the negligible leaching of manganese into the reaction mixture. Therefore, the prepared hybrid nanocatalyst has good reusability and stability.

## Conclusions

In this study, we have successfully synthesized an organic–inorganic hybrid nanostructure by immobilizing a Schiff base ligand onto an inorganic support material, followed by the immobilization of manganese (Fe_3_O_4_@SiO_2_@Mn-complex). The prepared hybrid nanostructure was thoroughly characterized. The utilization of this nanostructure allows for the efficient and selective synthesis of pharmaceutically interesting functionalized 7-arylchromeno[4,3-*d*]pyrido[1,2-*a*]pyrimidin-6-ones through a one-pot three-component reaction involving 4-hydroxycoumarin, substituted aromatic aldehydes, and 2-aminopyridine in aqueous ethanol under ambient conditions. The desired products were obtained in high yields and within short reaction times. This hybrid nanostructure exhibits high catalytic activity due to the synergistic effects among the organic ligand, Lewis acid, and porosity of the inorganic support. The notable advantages of this protocol include the use of aqueous ethanol as a green solvent, operational simplicity, energy efficiency, short reaction times, high to excellent yields, high atom-economy, easy separation of the catalyst, reusability, and elimination of the need for tedious column chromatography during product isolation and purification. These aspects align with the principles of green and sustainable chemistry. The interaction between the organic ligand and the inorganic support material creates a distinct microenvironment that enhances catalytic efficiency and selectivity.

## Author contributions

M. A. Bodaghifard: conceptualization, supervision, formal analysis, writing – review & editing. S. A. Pourmousavi: funding acquisition, project administration, validation. N. Ahadi: methodology, formal analysis, visualization, writing the original draft. P. Zeynali: investigation, methodology, data curation. All authors approved the final version of the manuscript to be published.

## Conflicts of interest

There are no conflicts to declare.

## Supplementary Material

NA-006-D4NA00131A-s001
