# Supplementary material for: An immobilized Schiff base–Mn complex as a hybrid magnetic nanocatalyst for green synthesis of biologically active [4,3-d]pyrido[1,2-a]pyrimidin-6-ones
Source: Nanoscale Adv. 2024 Apr 4;6(10):2713–21. doi: 10.1039/d4na00131a (PMC11093261; doi:10.1039/d4na00131a)
Supplement: NA-006-D4NA00131A-s001 [file NA-006-D4NA00131A-s001.pdf]

Electronic Supplementary Material (ESI) for Nanoscale Advances.
This journal is © The Royal Society of Chemistry 2024

Immobilized Schiff base-Mn complex as a hybrid magnetic nanocatalyst for green synthesis of biologically active [4,3-*d*]pyrido[1,2-*a*]pyrimidin-6-ones

Mohammad Ali Bodaghifard[*a,b], Seied Ali Pourmousavi[c], Najmieh Ahadi[b], Payam Zeynali[c]

[a]*Department of Chemistry, Faculty of Science, Arak University, Arak 384817758, Iran.*

[b]*Institute of Nanosciences &Nanotechnology, Arak University, Arak, Iran.*

[c]*School of Chemistry, Damghan University, Damghan 36716-45667, Iran*

**Correspondence to: Mohammad Ali Bodaghifard, E-mail: mbodaghi2007@yahoo.com; m-bodaghifard@araku.ac.ir*

^{1}H NMR and ^{13}C NMR spectral data for selected compounds

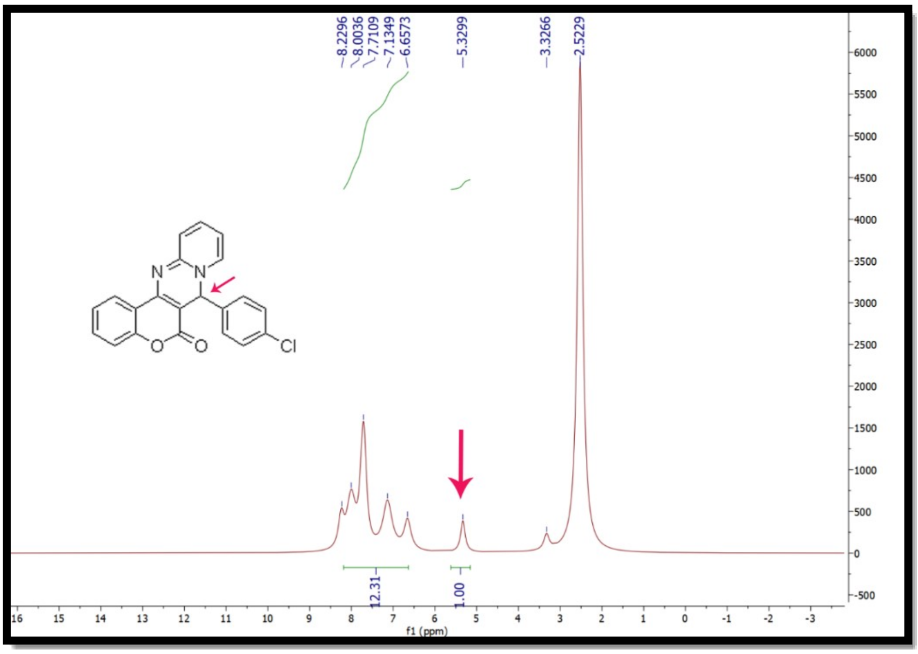

Fig. S1. ^{1}H NMR spectrum of 4a in DMSO-d_6

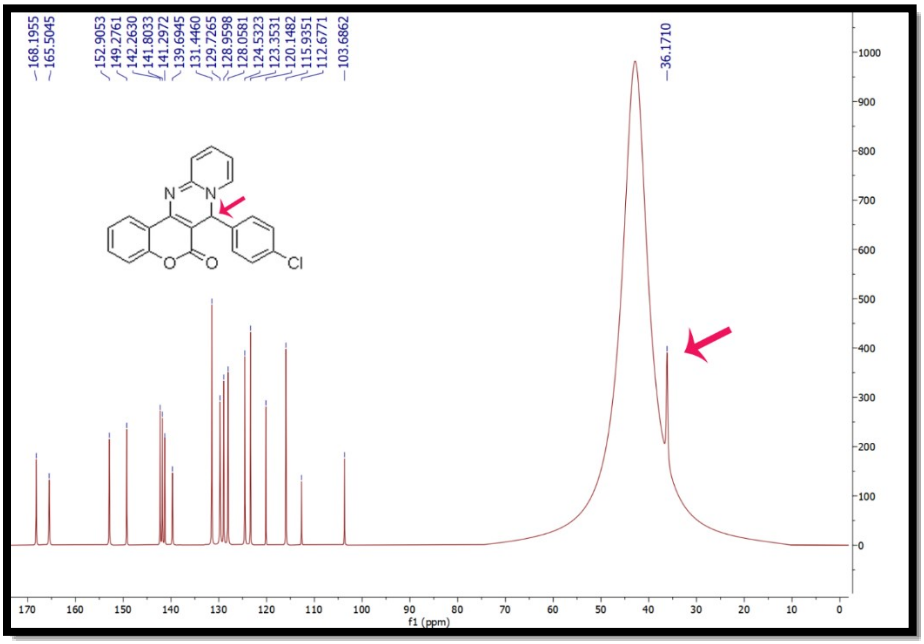

Fig. S2. ^{13}C NMR spectrum of 4a in DMSO-d_6

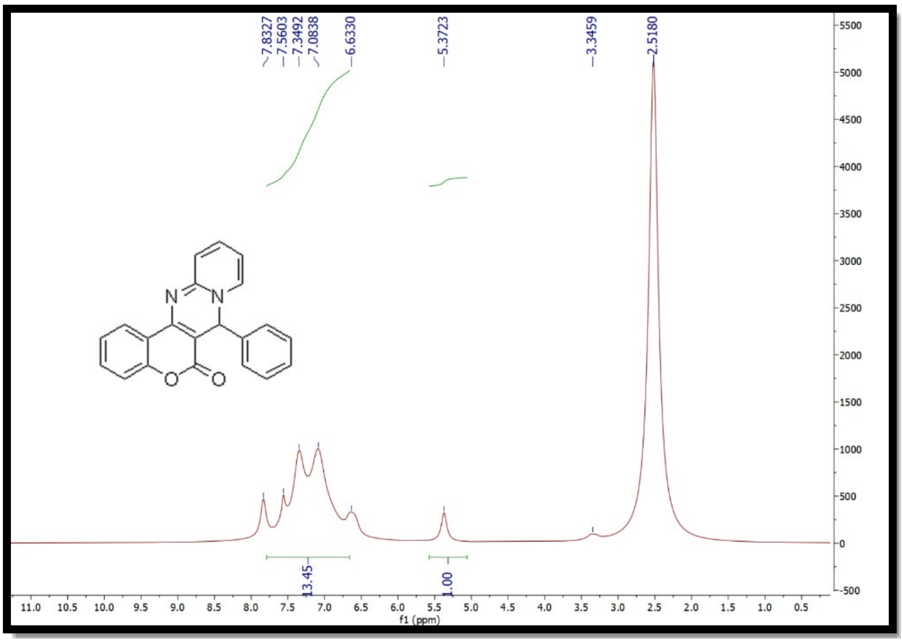

Fig. S3. ^{1}H NMR spectrum of 4b in DMSO-d_6

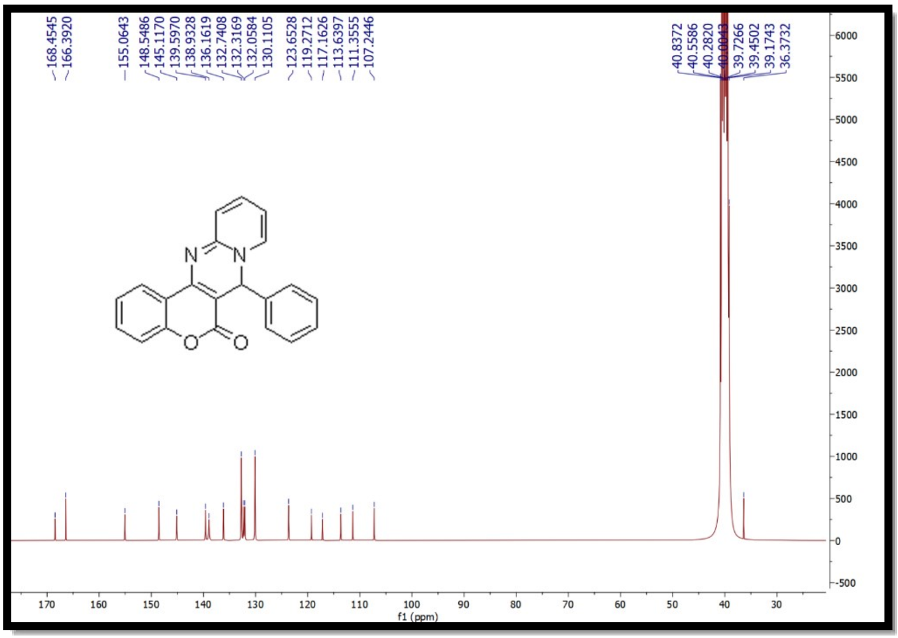

Fig. S4. ^{13}C NMR spectrum of 4b in DMSO-d_6

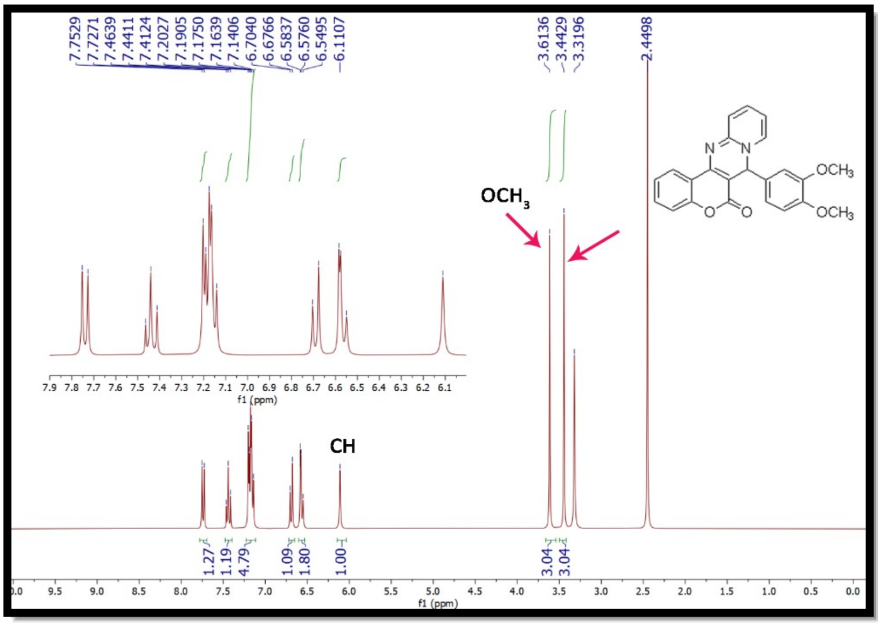

Fig. S5. ^{1}H NMR spectrum of 4h in DMSO-d_6

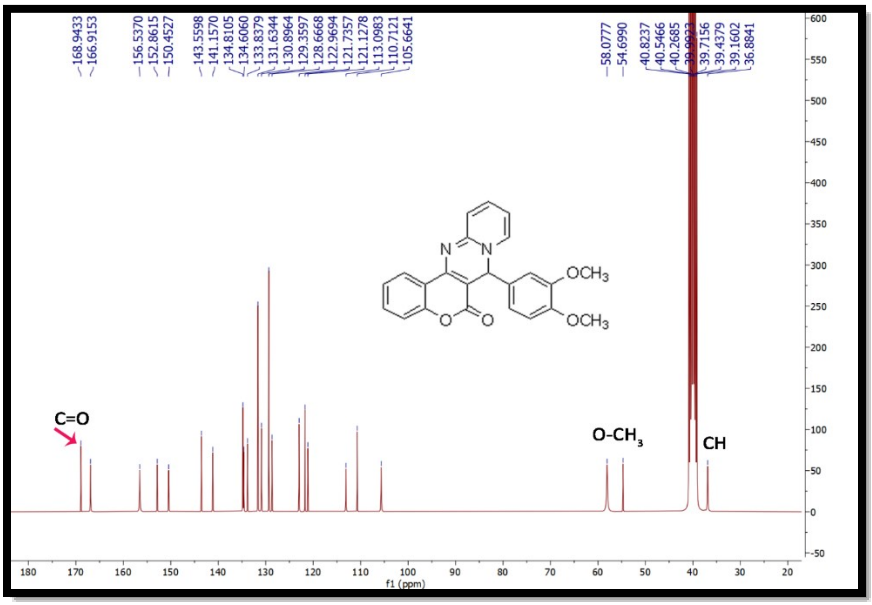

Fig. S6. ^{13}C NMR spectrum of 4h in DMSO-d_6

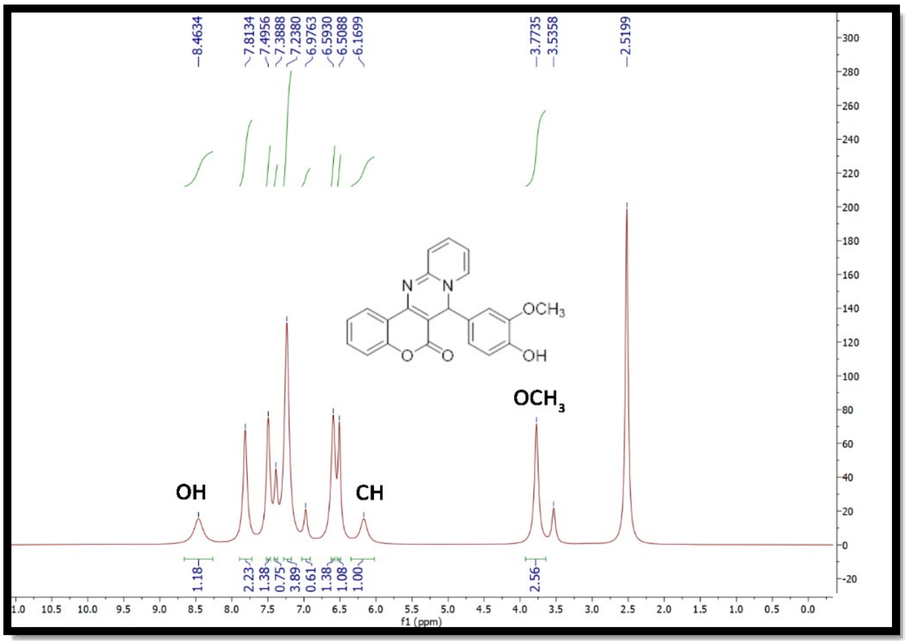

Fig. S7. ^{1}H NMR spectrum of 4j in DMSO-d_6

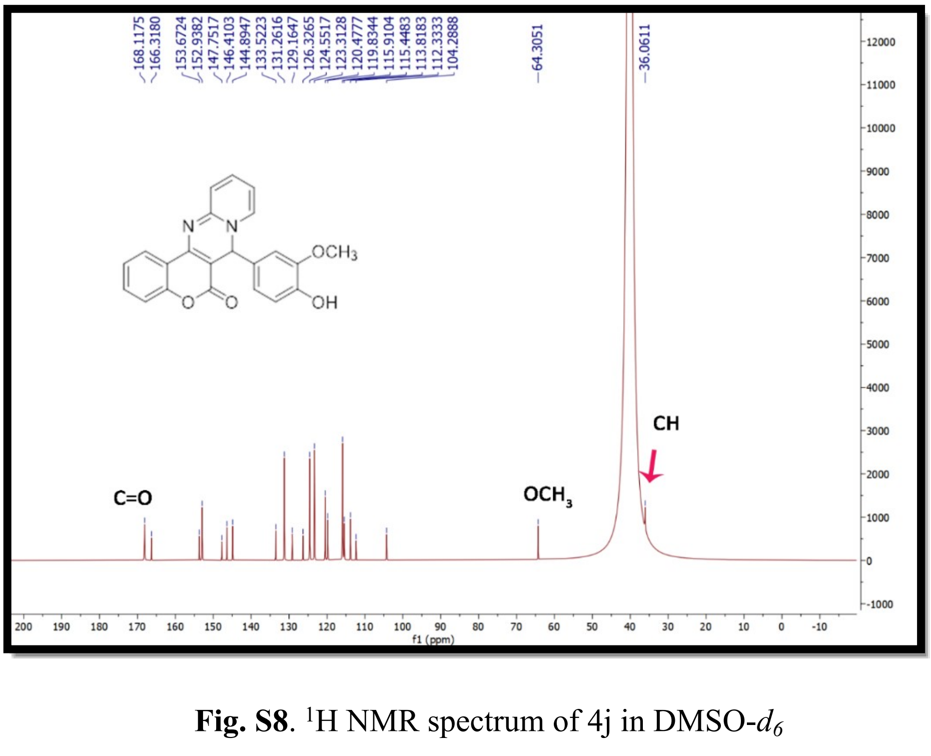

Fig. S8. ^{1}H NMR spectrum of 4j in DMSO-d_6

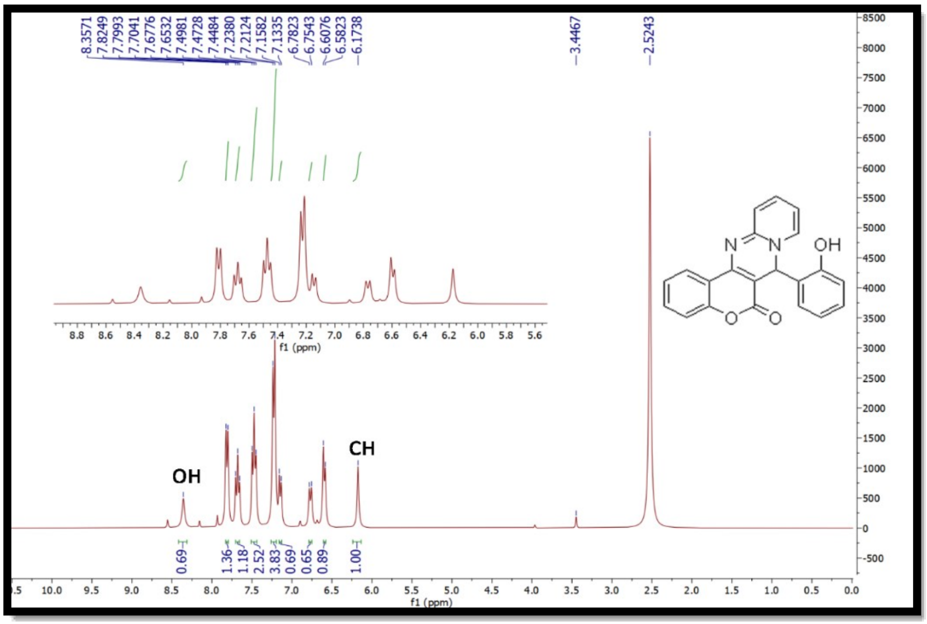

Fig. S9. ^{1}H NMR spectrum of 4l in DMSO-d_6

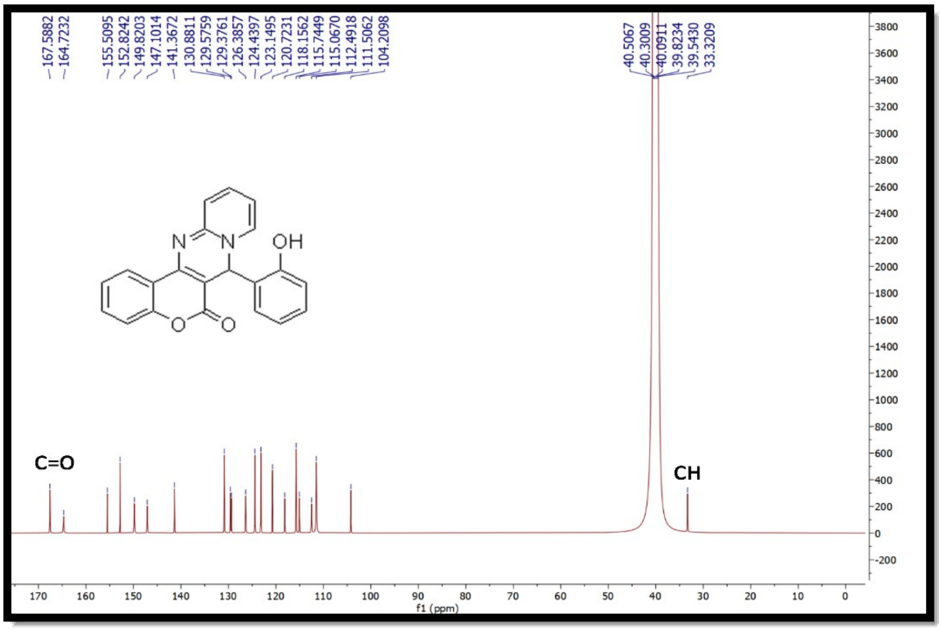

Fig. S10. ^{13}C NMR spectrum of 4l in DMSO-d_6

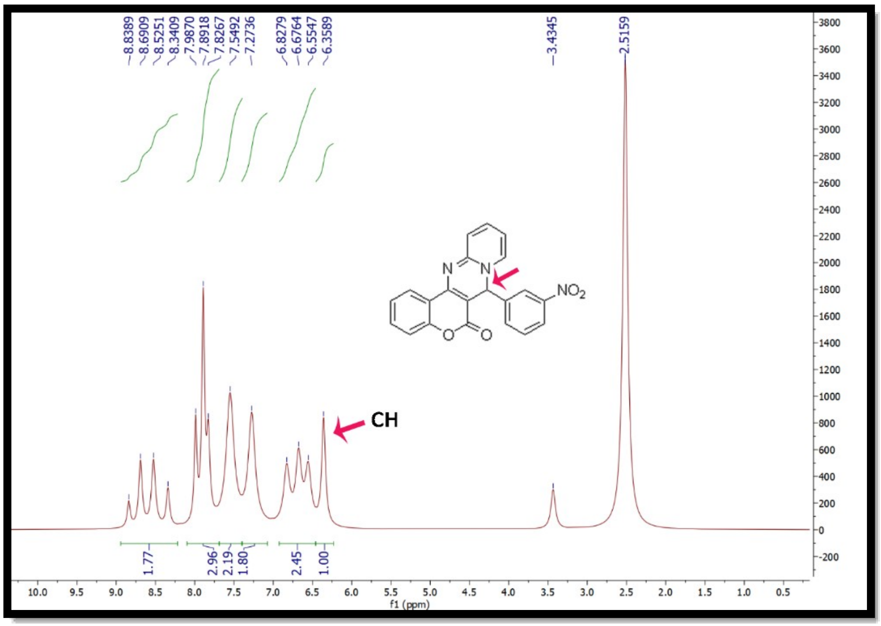

Fig. S11. ^{1}H NMR spectrum of 4m in DMSO-d_6

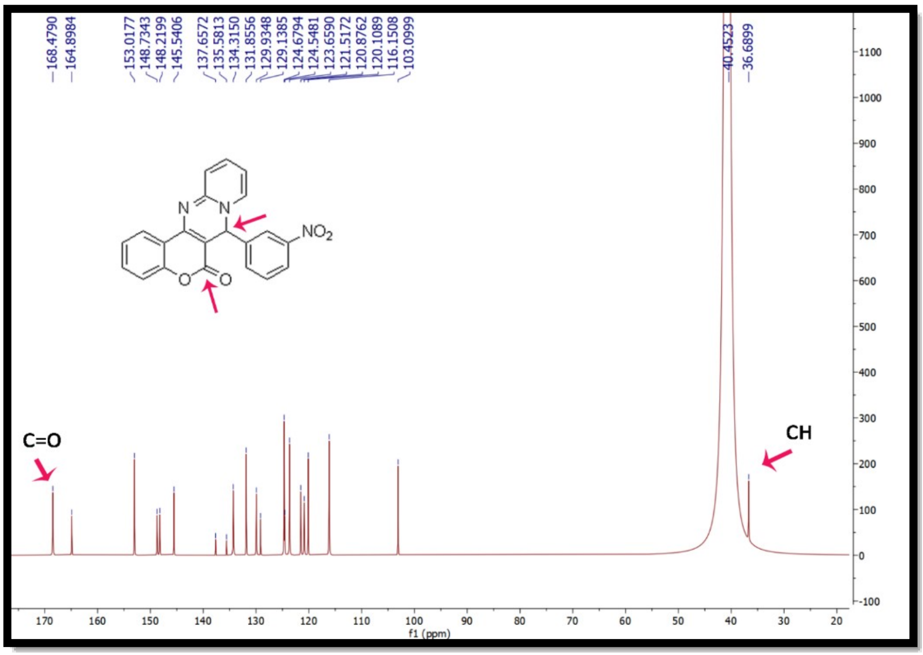

Fig. S12. ^{13}C NMR spectrum of 4m in DMSO-d_6
